# Justifying score interpretation thresholds for the Asthma Control Questionnaire (ACQ-5) in a contemporary asthma patient population

**DOI:** 10.1186/s41687-026-01097-y

**Published:** 2026-05-30

**Authors:** George Skingley, Mattea Orsini, Jessica Middlehurst, Jessica Flynn, James Brookes, Hermione Blackwood, Nathan Clarke, Rebecca Williams-Hall, Benoit Arnould, Renata Martincova, Jane Wells

**Affiliations:** 1 Patient Centered Outcomes, Adelphi Values, Bollington, Cheshire, UK; 2 Aixial, Sèvres, France; 3 Sanofi, Lyon, France; 4 Sanofi, Prague, Czech Republic; 5 Sanofi, Reading, UK

**Keywords:** Asthma control questionnaire, Asthma, Meaningful between-group difference, Meaningful within-patient change, Psychometric validation, Patient-reported outcomes

## Abstract

**Background:**

The 5-item Asthma Control Questionnaire (ACQ-5) is widely used in clinical trials to assess the adequacy of asthma symptom control and any changes in asthma control as a result of treatment. Evidence supporting the psychometric properties of the ACQ-5, including a 0.5-threshold for meaningful improvement, was derived using methods that are no longer recommended in the literature or by regulatory authorities. The primary objective of this research was to derive meaningful change estimates using contemporary methods to evaluate clinically important change for the ACQ-5. A secondary objective was to evaluate the applicability of the existing ACQ-5 asthma control threshold for defining “well-controlled” asthma (ACQ-5 ≤ 0.75).

**Methods:**

Measurement properties (item performance, reliability, validity, and responsiveness) were assessed prior to evaluating meaningful change, using data from a sample of 196 adults with inadequately controlled moderate-to-severe asthma from a placebo-controlled, Phase 2 study of rilzabrutinib (NCT05104892). Anchor- and distribution-based analyses were used to derive meaningful within-patient change (MWPC) and meaningful between-group difference (MBGD). Applicability of the existing ACQ-5 asthma control threshold was evaluated by plotting the positive predictive value (PPV) and negative predictive value (NPV) of the ACQ-5 total score in the current study against the PPV and NPV calculated in the original Juniper et al. (2006) study.

**Results:**

High internal consistency (Cronbach’s alpha = 0.90), moderate-to-strong convergent validity (r range: 0.52–0.83), and ability to detect improvements in asthma control based on psychometric and physiological measures were demonstrated for the ACQ-5. Test-retest reliability results were poor-to-moderate (intraclass correlation coefficient range: 0.30–0.66). Of the anchor-based estimates derived (0.2 to 1.6), empirical cumulative distribution function plots supported 0.6 or 0.8 as appropriate MWPC thresholds. Estimated MBGD ranged from 0.2 to 0.6. PPV/NPV plots supported continued use of a 0.75- threshold to determine “well-controlled” asthma.

**Conclusion:**

Contemporary score interpretation methods support a MWPC threshold of ≥ 0.6, which is functionally identical to the extant threshold of ≥ 0.5, given the 0.2 increments of the ACQ-5 scale. MBGD ranged from 0.2 to 0.6, further supporting use of the established ≥ 0.5 threshold to define clinically important change between groups of patients. A “well-controlled” threshold of ≤ 0.75 was also supported.

**Supplementary Information:**

The online version contains supplementary material available at 10.1186/s41687-026-01097-y.

## Introduction

Asthma is a chronic inflammatory lung disease, characterized by recurrent episodes of breathlessness, wheezing, chest tightness, and cough, which make it difficult to breathe [1, 2]. It is one of the most prevalent chronic, non-communicable diseases, affecting an estimated 334 million people worldwide [3]. Asthma symptoms can vary over time and intensity, with variable expiratory airflow limitation [1, 2]. Symptoms may resolve spontaneously or in response to treatment [1, 2].

The long-term goals of asthma management are to achieve good symptom control (e.g., few or no asthma symptoms, unimpaired physical activity, no sleep disturbances) and minimize asthma-associated risks (e.g., future exacerbations, fixed airflow limitation, maintenance systemic corticosteroids, asthma-related deaths) [1]. Treatment options include inhaled corticosteroids-containing medication (ICS) in isolation, when taking a short-acting beta antagonist, or in combination with a long-acting beta-agonist (LABA). Reliever inhalers for symptom relief and add-on controller medications are also available for patients with severe disease [1]. Patients with severe asthma that is inadequately controlled using standard inhaled or oral medications may also be prescribed biologic therapies to help improve disease control [4]. Despite these available therapies, a large proportion of patients experience inadequately controlled asthma [4–6].

The 5-item Asthma Control Questionnaire (ACQ-5) is a patient-reported outcome (PRO) measure designed to assess the adequacy of asthma symptom control and changes in symptom control as a result of treatment [7]. The ACQ-5 has frequently been used to support asthma clinical trial endpoints to demonstrate changes in asthma symptom control following treatment [8]. To assess such changes, an understanding of the amount of change in ACQ-5 scores that is meaningful to patients is needed. A meaningful improvement threshold of ≥ 0.5 has been proposed in the literature [9] and has been used to support endpoints in asthma trials [10–14]. However, recent developments within the score interpretation literature have led to contemporary recommendations to derive both within-individual and between-group thresholds [15], and currently there is ambiguity as to whether the extant 0.5 threshold is applicable to between-group difference and meaningful within-patient change (MWPC) [11, 16, 17].

Further to this ambiguity, there are various contemporary methodological recommendations pertaining to derivation of score interpretation thresholds that warrant revisiting the extant 0.5 threshold for the ACQ-5. The 0.5 threshold was established solely using a regression-based method (i.e., geometric mean regression) [9], with contemporary recommendations preferring methods such as receiver operating characteristic (ROC) curves to derive MWPC estimates [15]. Further, the Mini Asthma Quality of Life Questionnaire [18], a health-related quality of life (HRQoL) instrument, was used as the anchor to define meaningful change [9]; however, current guidance recommends the use of measures that allow meaningfulness to be directly inferred, such as by using a patient global impression of severity (PGIS) item [19]. Contemporary score interpretation literature also advocates that within-patient thresholds should reflect change that is achievable given the scale increments [20] and that ranges should be provided for between-group thresholds [19]. Given recent developments in contemporary score interpretation literature, there is a need to re-evaluate meaningful change estimates for the ACQ-5 using contemporary methodology.

Within the context of a clinical trial, diagnostic classification thresholds enable researchers to identify patients who will benefit most from an intervention (i.e., those who are inadequately controlled), as well as define treatment success between groups of patients. Well-controlled asthma has previously been defined as an ACQ-5 score ≤ 0.75, based on data collected during the 2004 Gaining Optimal Asthma Control (GOAL) clinical trial [21]. Similar to score interpretation thresholds, there is value in evaluating the applicability of this diagnostic classification threshold in a sample of contemporary asthma patients who more accurately reflect current unmet treatment needs.

The objectives of this research were to derive meaningful change estimates (primary aim) and confirm applicability of the existing “well-controlled” asthma threshold (secondary aim) to support clinical trial endpoints, and ultimately label claims awarded by regulatory health authorities. Measurement properties of the ACQ-5 were also assessed as a precursor to evaluating score interpretation thresholds.

## Methods

### Study design and sample

Treatment-blinded data from a Phase 2, randomized, double-blind, placebo-controlled, 12-week proof-of-concept (POC) study evaluating the efficacy, safety, and tolerability of rilzabrutinib in adults with moderate-to-severe asthma who were inadequately controlled on ICS plus LABA therapy (NCT05104892) were pooled across treatment groups to evaluate the measurement properties and derive score interpretation thresholds for the ACQ-5. The study design and eligibility criteria for the study have been previously described [22]. Patients aged 18 to 70 years with an ACQ-5 score between 1.25 and 3.00 at randomization were assigned to receive rilzabrutinib 400 mg or placebo oral tablets, administered twice daily (BID) or three times daily (TID). This investigational treatment was added to their existing background therapy of medium-to-high dose ICS/LABA initiated at screening, which was then withdrawn from weeks 4–9 and resumed at the end of the 12-week treatment period. Participants who experienced a loss of asthma control (LOAC) event during the randomized treatment period were discontinued early from treatment and withdrawn from the study.^1^ Participants were recruited from clinical sites in Argentina, Canada, Hungary, Korea, Mexico, Poland, Romania, Spain, Turkey, Ukraine, and United Kingdom. An institutional review board at each site approved the study protocol and all activities were conducted in compliance with the International Committee on Harmonization and applicable Good Clinical Practice standards and in accordance with the Declaration of Helsinki and its later amendments. All participants provided written informed consent.

### Measures

#### Asthma control questionnaire (ACQ-5)

The ACQ-5 consists of five questions, in the following order, assessing frequency of nighttime awakenings, severity of symptoms upon waking, severity of impact on daily activities, severity of shortness of breath, and frequency of wheeze [7]. Participants are asked to recall how their asthma has been “on average” (Items 1–2) or “in general” (Item 3–5) “during the past week”, using a 7-point verbal rating scale ranging from 0=“no impairment” to 6=“maximum impairment”. The ACQ-5 score is the mean of the five questions with higher scores indicating low asthma control (ranging from 0 to 6). In the study, the ACQ-5 was completed on an electronic device at Screening, Baseline, and Weeks 2, 4, 6, 8, 10, and 12.

#### Concurrent measures

Other clinician- and patient-reported outcome measures administered alongside the ACQ-5 in the study were used to support evaluation of convergent validity of the ACQ-5, define patients with stable asthma for test-retest reliability analysis, and define subjects who experienced change, detailed in Table 1.

**Table 1:** Convergent validity measures

Assessment	Description
Patient Global Impression of Severity (PGIS)	This PGIS is a single item that asks patients to provide a self-assessment of their overall asthma severity for the past week on a 4-point scale (0=“none”, 1=“mild”, 2=“moderate”, 3=“severe”). The PGIS was administered at Baseline and Weeks 4, 8, and 12.
Patient Global Impression of Change (PGIC)	The PGIC is a single item that asks patients to provide a self-assessment of overall change in their asthma severity since starting the study medication on a 7-point Likert scale (0=“very much better”, 1=“moderately better”, 2=“a little better”, 3=“no change”, 4=“a little worse”, 5=“moderately worse”, 6=“very much worse”). The PGIC was administered at Weeks 4, 8, and 12.
Asthma Daytime Symptom Diary (ADSD) and Asthma Nighttime Symptom Diary (ANSD)	The ADSD and ANSD are patient-reported outcome measures developed to assess asthma symptoms in patients 12 years and older with mild-to-severe asthma. Each measure assesses asthma severity based on patients’ self-report of difficulty of breathing, wheezing, shortness of breath, chest tightness, chest pain, and cough at their worst. Patients complete the ADSD every night before bed (thinking about their asthma symptoms that day) and the ANSD every morning when they get up (thinking about their asthma symptoms the previous night). Each item is scored on an 11-point numeric rating scale (0=“none” and 10=“as bad as you can imagine”). Average total scores are derived for both diaries separately, with higher scores indicating more severe asthma symptoms. Scores can only be calculated if data for four or more items are available, with the available item values summed and divided by the number of items available [23]. The ADSD and ANSD were administered daily as part of an electronic diary.
Spirometry Assessments	Forced Expiratory Volume in one second (FEV1) [24] and Peak Expiratory Flow (PEF) [25] were performed in accordance with the American Thoracic Society (ATS)/European Respiratory Society (ERS) guidelines [26]. FEV1 and PEF were conducted each week in the morning following a wash out period of bronchodilators.
Electronic diary/PEF meter	An electronic diary/PEF meter was used to record daily levosalbutamol/levoalbuterol or salbutamol/albuterol use, including the number of inhalations per day for symptom relief. Participants were instructed to try to withhold from using albuterol or levalbuterol for at least six hours prior to measuring PEF.

### Psychometric methods

The psychometric analysis population, comprised of all randomized participants with non-missing Baseline or post-Baseline data for the ACQ-5, was used for all analyses. All analyses were pre-specified in a psychometric analysis plan and conducted in accordance with best practice guidance from regulators [19, 27, 28].

#### Measurement properties of the ACQ-5

##### Item properties

Missing data at the form-level and distributions of responses to each ACQ-5 item were summarized at Baseline and Weeks 4, 8, and 12 to assess quality of completion and identify any response options that were over- or under-endorsed, or that demonstrated a floor (a high percentage scoring the worst possible score) or ceiling (a higher percentage scoring the best possible score) effect.

##### Internal consistency and test-retest reliability

Internal consistency was assessed by calculating Cronbach’s alpha coefficient (α ≥ 0.70 considered evidence of acceptable internal consistency reliability) [29] using data from Baseline and Week 12. Test-retest reliability was evaluated by examining the intraclass correlation coefficients (ICCs) for participants defined as “stable” based on the Patient Global Impression of Severity (PGIS) from Baseline to Week 4 and from Week 8 to Week 12. ICC values < 0.50 were considered as indicating poor reliability, 0.50–0.75 as indicating moderate reliability, 0.75–0.90 as indicating good reliability, and > 0.90 as indicating excellent reliability [30].

##### Construct validity evidence

Convergent validity was evaluated by examining ACQ-5 correlations with the PGIS, Asthma Daytime Symptom Diary (ADSD), and Asthma Nighttime Symptom Diary (ANSD) at Baseline and Week 12. All convergent measures were expected to correlate > 0.50 with the ACQ-5. The known-groups method explored how scores at Baseline and Week 12 differed among patients on variables hypothesized to influence the construct of interest (PGIS [0=“none”, 1=“mild”, 2=“moderate”, 3=“severe”] and reliever medication use [mean inhalations < 1/day vs. ≥ 1/day]). Differences in mean scores between groups were tested using F-test with one-way ANOVA (*p* ≤ 0.05 considered statistically significant). Between-group effect sizes (ESs) were calculated to determine the magnitude of difference (small: ES = 0.20, moderate: ES = 0.50, large: ES = 0.80) [31].

##### Ability to detect change

Ability to detect change was assessed by evaluating mean change in scores from Baseline to Week 12 in groups categorized as “improved” (i.e., any improvement), “stable” (i.e., no change), or “worsened” (i.e., any worsening) according to the PGIS, Patient Global Impression of Change (PGIC), Forced Expiratory Volume (FEV1), and PEF in separate analyses. Within-group ESs [32] (i.e., mean change score divided by the standard deviation of the score at the earlier of two timepoints) were calculated to evaluate the magnitude in change scores within each group (small: ES = 0.20, moderate: ES = 0.50, large: ES = 0.80) [31]. Between-group ES were calculated using the same method as for known-groups. One-way ANOVA F-tests were used to evaluate the statistical significance of differences in change scores between each group.

#### Interpretation of scores

##### Anchor-based analyses

Potential anchors for this analysis were the PGIS, PGIC, FEV1, PEF, and reliever medication. Cross-sectional and change from Baseline correlations were calculated between the anchors and ACQ-5 at Baseline (cross-sectional only), Week 4, Week 8, and Week 12. The resultant correlations were Fisher’s Z-transformed and then averaged. Only anchors correlating ≥|0.3| in magnitude were taken forward for the meaningful change analyses [33].

###### Meaningful within-patient change (MWPC)

MWPC estimates were derived using mixed models for repeated measures (MMRMs) [34], incorporating all data available from Baseline to Week 12. Each model had the change from Baseline score for the ACQ-5 as the dependent variable and the anchor change as a categorical, independent variable. The interaction term of visit and anchor was also estimated, allowing mean differences to vary by visit. Anchor groups containing fewer than 10 participants were excluded. The MWPC estimate was defined as the least squares (LS) mean difference between the ‘minimally improved’ group minus the ‘stable’ group. Standardized between-group ES were calculated as the mean difference divided by the standard deviation of the ACQ-5 score at Baseline. Estimates considered negligibly small or untenably large according to their standardized ES (i.e., < 0.2 or ≥ 2.0) were not taken forward for triangulation.

Receiver operating characteristic (ROC) curves were used to identify the change score that optimally discriminated between all “improved” and “stable” groups. Empirical cumulative distribution function (eCDF) plots were also produced to assess the suitability of possible thresholds by examining the functions of different change groups on each anchor [35].

###### Meaningful between-group difference (MBGD)

MBGD estimates were derived using MMRMs with the ACQ-5 score at each visit as the dependent variable and the anchor score as the independent variable. The interaction term of visit and anchor was also estimated, allowing mean differences to vary by visit. The LS mean differences between the anchor group scores from the MMRM were used to inform MBGD (e.g., ‘None’ minus ‘Mild’ group) [34]. Mean change scores were also calculated to determine MBGD using data from Baseline to Week 4, Week 8, and Week 12 [36]. The MBGDs were estimated by comparing the mean of each anchor change group to the stable group.

##### Distribution-based analyses

Distributional properties of the ACQ-5 were used to provide an indication of the amount of change beyond measurement error that may be considered meaningful. Estimates were calculated as 0.5 of the standard deviation (SD) at Baseline and the standard error of measurement (SEM) [15, 37–39]. The SEM was calculated as the SD at Baseline multiplied by the square root of one minus the reliability of the score at Baseline [$\:SD*{(1-r)}^{1/2}$].

##### Triangulation of estimates

MWPC estimates were visualized on a forest plot to aid triangulation and indicate concordance of estimates. Possible thresholds were subsequently evaluated with eCDFs and triangulated with consideration of SEM, the actual attainable score increments for individual patients, and score interpretation thresholds proposed in previous research [9]. Existing score interpretation thresholds were also considered for the MBGDs [9].

##### Classification statistics analysis evaluating ACQ-5 ‘well-controlled’ asthma threshold

An analysis was conducted to evaluate the applicability of the existing ACQ-5 asthma control threshold for defining “well-controlled” asthma (ACQ-5 score ≤ 0.75). For this analysis, the methods used to derive the existing threshold were replicated to the extent feasible [21] and in consideration of the updated GINA guidelines for defining asthma control (i.e., absence of daytime and nighttime symptoms, minimal or no need for reliever medication, no limitations on physical activity, normal or near-normal lung function, and avoidance of severe exacerbations) [1] collected via proxy items as part of an electronic case report form (see Table S1). The positive predictive value (PPV) and negative predictive value (NPV) for the ACQ-5 total score for “well-controlled” asthma were then calculated, using data pooled across timepoints (i.e., Baseline, Week 4, Week 8, and Week 12). The resulting PPV and NPV were plotted against the PPV and NPV calculated in the original study [21] to identify the point at which these intersected.

## Results

### Sample characteristics

All 196 patients randomized were included in the psychometric analysis population. Key demographic and clinical characteristics are provided in Table 2. The sample had a mean age of 48.9 years (range: 18–70 years) and most were white (95.4%), female (62.2%), and had never smoked (87.8%). Mean ACQ-5 score at Baseline was 2.18, with higher scores indicative of worse asthma control.

**Table 2:** Demographic and clinical characteristics for the psychometric analysis population at baseline

Characteristic	Psychometric Analysis Population (*n* = 196)	
**Age (years)**		
Mean (SD)	48.9 (13.30)	
Median	50.0	
Q1; Q3	40.0; 61.0	
Min; Max	18; 70	
**Gender**,** n (%)**		
Female	122 (62.2)	
Male	74 (37.8)	
**Region**,** n (%)**		
Eastern Europe	95 (48.5)	
Latin America	88 (44.9)	
Western Countries	7 (3.6)	
Asia and Pacific Region	6 (3.1)	
**Race**,** n (%)**		
White	187 (95.4)	
Asian	6 (3.1)	
American Indian or Alaska Native	2 (1.0)	
Black or African American	1 (0.5)	
**Ethnicity**,** n (%)**		
Not Hispanic or Latino	121 (61.7)	
Hispanic or Latino	75 (38.3)	
**Body weight (kg)**		
Mean (SD)	79.2 (13.76)	
Median	78.9	
Q1; Q3	71.0; 89.0	
Min; Max	48.0; 126.0	
**Body Mass Index (BMI**,** kg/m2)**		
Mean (SD)	28.3 (4.51)	
Median	27.6	
Q1; Q3	25.1; 31.0	
Min; Max	19.6; 40.1	
**Smoking history**,** n (%)**		
Never	172 (87.8)	
Former	24 (12.2)	
**Atopic medical condition**,** n (%)**		
No	114 (58.2)	
Yes	82 (41.8)	
**Age at onset of asthma**,** n (%)**		
< 12	45 (23.0)	
≥ 12 ≤ 18	17 (8.7)	
≥ 18 ≤ 40	84 (42.9)	
≥ 40	50 (25.5)	
**ACQ-5 (the lower the better – range 0 to 6)**		
Mean (SD)	2.18 (0.43)	
Median	2.20	
Q1; Q3	1.90; 2.40	
Min; Max	1.20; 4.00	
Pre-bronchodilator forced expiratory volume in the first second (FEV-1) (%)*		
Mean (SD)	73.7 (15.37)	
Median	74.0	
Q1; Q3	62.0; 83.0	
Min; Max	35.0; 118.0	
**Peak Expiratory Flow (PEF) (L/min)**	AM**	PM***
Mean (SD)	313.5 (118.43)	319.8 (115.09)
Median	295.3	306.3
Q1; Q3	224.7; 382.4	232.6; 381.0
Min; Max	96.5; 632.4	99.7; 651.6

Abbreviations: ACQ-5 = 5-item Asthma Control Questionnaire; N = Number of participants; SD = Standard Deviation; Q1 = 25th percentile; Q3 = 75th percentile; BMI = Body Mass Index; FEV-1 = Forced expiratory volume in 1 s; PEF = Peak Expiratory Flow

*n = 194; **n = 195; ***n = 193

### Measurement properties of the ACQ-5

#### Item properties

The quality of completion of the ACQ-5 was high across all timepoints (≥ 83.1%). No floor effects were observed at any timepoint; however, ceiling effects > 15% [40] were consistently observed for Item 1 (Frequency of nighttime awakenings), Item 3 (Impact on daily activities), and Item 5 (Frequency of wheeze) at all post-Baseline timepoints (see Figures S1-S4).

#### Internal consistency and test-retest reliability

Internal consistency was excellent at Week 12 (Cronbach’s α = 0.90, 95% CI = 0.87,0.92) but below the standard acceptability threshold of ≥ 0.70 at Baseline (α = 0.61, 95% CI = 0.51,0.89). Calculation of alpha-if-item-deleted coefficients at both timepoints (Baseline range: 0.52–0.60; Week 12 range: 0.86–0.89) further supported internal consistency of ACQ-5 items (Table S2).

Test-retest reliability results for participants defined as stable based on the PGIS were poor from Baseline to Week 4 (*n* = 98; ICC = 0.30, 95% CI = 0.01,0.52) and moderate from Week 8 to Week 12 (*n* = 93; ICC = 0.66, 95% CI = 0.53,0.76).

#### Construct validity

All correlations between the ACQ-5 scores and concurrent measures (i.e., PGIS, ADSD, and ANSD) were below the hypothesized threshold of > 0.50 at Baseline but were moderate-to-strong (*r* = 0.52 to 0.83) at Week 12, providing evidence of convergent validity (Table S3).

Known-groups comparisons demonstrated statistically significant differences in ACQ-5 scores between groups defined by PGIS severity at Baseline and Week 12 (*p* < 0.001). Monotonically increasing scores across severity groups were identified and ESs indicated differences between adjacent groups were moderate to large (Baseline: ES = 0.56–0.81; Week 12: ES = 1.43–2.42), except for those categorized as “mild” at Baseline (ES = 0.16). A statistically significant difference (*p* < 0.001) and large ES (ES = 0.96) was also observed at Week 12 comparing mean ACQ-5 scores by participants with an average of ≥ 1/<1 inhalations of reliever medication per day. Results were generally poorer at Baseline (Table S4).

#### Ability to detect change

Changes in ACQ-5 scores were compared among participants defined as “improved”, “stable”, and “worsened” on the PGIS, PGIC, FEV1, and PEF separately from Baseline to Week 12 (Table 3). The ACQ-5 indicated ability to detect improvement, with large within-group ESs (ES range: -1.99 to -2.47 in magnitude) for the improved group for all anchors; in all cases, the ES for the stable group was smaller than the improved group. Between-group ESs were moderate for the PGIS (ES = 0.64) and PEF (ES = 0.53) and small for the PGIC (ES = 0.30) and FEV1 (ES = 0.20) for mean change between the improved and stable groups.

**Table 3:** Ability to detect change of the ACQ-5 from baseline to week 12 in the psychometric analysis population

Anchor measure	Anchor category	*n*	Median	Within-Group mean change score (95% CI)	Within-Group effect size	Between-Group mean change score (95% CI)	*P*-value	Between-Group effect size
PGIS	Any improvement	68	-1.00	-0.97 (-1.14, -0.80)	-2.28	-0.47 (-0.73, -0.21)	< 0.0001	0.64
	Stable: No change	56	-0.40	-0.50 (-0.71, -0.29)	-1.17	-	-	-
	Any worsening	12	-0.10	0.13 (-0.76, 1.02)	0.31	0.63 (0.04, 1.22)	-	0.06
PGIC	Any improvement	113	-0.80	-0.85 (-0.99, -0.70)	-1.99	-0.23 (-0.65, 0.18)	< 0.0001	0.30
	Stable: No change	15	-0.40	-0.61 (-1.05, -0.17)	-1.44	-	-	-
	Any worsening	9	0.60	0.51 (-0.05, 1.08)	1.23	1.12 (0.45, 1.80)	-	1.45
FEV1	Any improvement	29	-1.20	-0.99 (-1.35, -0.62)	-2.31	-0.16 (-0.58, 0.26)	0.0335	0.20
	Stable: No change	35	-0.80	-0.82 (-1.07, -0.57)	-1.93	-	-	-
	Any worsening	59	-0.40	-0.48 (-0.74, -0.23)	-1.14	0.34 (-0.04, 0.71)	-	0.38
PEF	Any improvement	30	-1.20	-1.05 (-1.37, -0.74)	-2.47	-0.49 (-0.91, -0.06)	0.0498	0.53
	Stable: No change	47	-0.60	-0.57 (-0.85, -0.29)	-1.33	-	-	-
	Any worsening	51	-0.60	-0.62 (-0.86, -0.37)	-1.44	-0.05 (-0.42, 0.32)	-	0.05

*Abbreviations*: CI=Confidence Interval; FEV=Forced Expiratory Volume; PEF=Peak Expiratory Flow; PGIC=Patient Global Impression of Change; PGIS=Patient Global Impression of Severity

Few participants worsened on PGIS and PGIC (*n* = 9 and *n* = 12 respectively), precluding interpretation of ability to detect worsening. PEF showed the same level of mean change for worsened and stable groups while FEV1 showed a small between-group ES indicating worsening (ES = 0.38).

### Interpretation of scores

#### Anchor correlations

Anchor correlations at Week 12 between change in ACQ-5 scores and both the PGIS and PGIC items showed that they were sufficiently correlated (*r* > 0.40), and both were taken forward for subsequent meaningful change analyses; no other anchors were taken forward. Cross-sectional and change from Baseline correlations (averaged across timepoints) for potential anchor measures are presented in Table 4.

**Table 4:** Correlations between ACQ-5 and anchors

Anchor	Averaged correlation coefficient	
	Mean cross-sectional correlation	Mean change from baseline correlation
PGIC	─	**0.451**
PGIS	**0.722**	**0.400**
FEV1	-0.028	-0.193
PEF	-0.048	-0.151
Reliever medication	0.144	0.192

*Abbreviations*: FEV=Forced Expiratory Volume; PEF=Peak Expiratory Flow; PGIC=Patient Global Impression of Change; PGIS=Patient Global Impression of Severity

*Note: all cross-sectional correlations are Pearson’s*,* except those with PGIS*,* which are Polyserial. Change from Baseline correlations are Spearman’s (FEV1*,* PEF*,* reliever medication) and Polyserial (PGIC and PGIS). PGIC is a change score and was not calculated for cross-sectional analysis. Bolded text indicates the anchor was sufficiently correlated with the ACQ-5 to support the meaningful change analyses*

#### Meaningful within patient change threshold

##### Anchor-based estimates

A relatively wide range of values (-0.24 to -1.60) for potential ACQ-5 meaningful change thresholds were estimated across the available anchors and methods (Table 5), which were subsequently triangulated.

##### Distribution-based estimates

Half the standard deviation of the ACQ-5 at Baseline was 0.21. This was smaller than the SEM (0.36), which was used to inform likely measurement error of a MWPC threshold.

##### Triangulation of anchor-based estimates

**Fig. 1:**
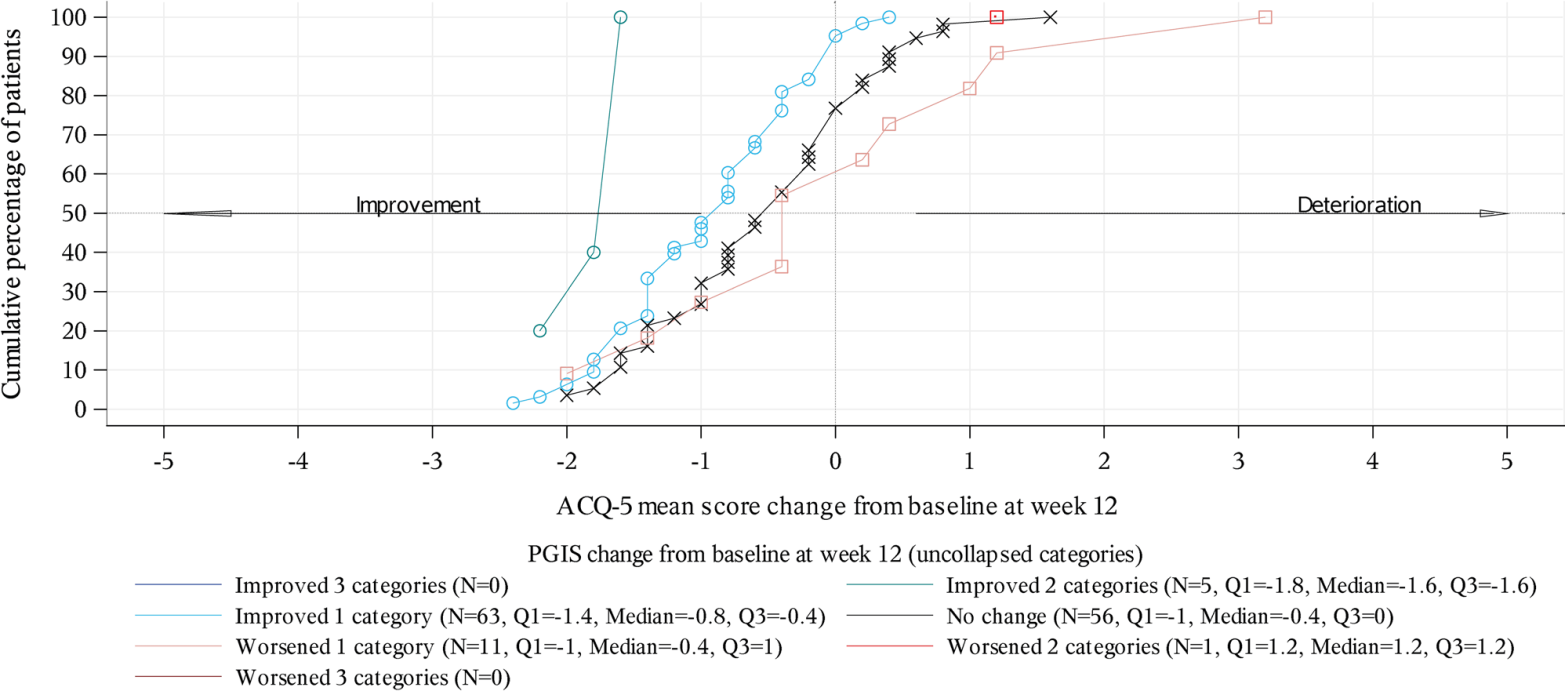
eCDF of ACQ-5 mean change from baseline to Week 12 by PGIS change category in the psychometric analysis population

Estimates were summarized on a forest plot to identify convergence around a small range of values (Figure S5). The PGIS estimates suggested a potential improvement of ≥ 0.4 at Week 12. Consultation of the eCDF plot, however, indicated a MWPC improvement of ≥ 0.4 would incorrectly classify around 55% of stable participants as improved (Fig. 1). A MWPC improvement of ≥ 0.6 (the next attainable change value on the scale) was therefore evaluated, which indicated < 50% of stable patients would be classified as improved and 68% of 1-category improved patients would be classified as improved. The only other viable threshold was a MWPC improvement of ≥ 0.8, which would classify approximately 40% of stable patients as improved and 60% of 1-category improved patients as improved. However, given the relatively equal performance of 0.6 and 0.8 on the eCDF plot, evidence generated in this study was not considered strong enough to disregard the existing 0.5 threshold (i.e., to support an alternative threshold of 0.8).

##### Proposed MWPC threshold from triangulation

Table 5 shows the estimates and resulting proposed MWPC threshold for improvement. A minimum MPWC threshold of ≥ 0.6 improvement was supported for the current analysis. A threshold of ≥ 0.6 was further supported by the distribution-based analyses (SEM = 0.36), suggesting this threshold would exceed measurement error. Given the ACQ-5 response scale has 0.2 increments, an improvement of ≥ 0.6 is functionally identical to the ≥ 0.5 within-individual threshold proposed in the existing literature [9].

**Table 5:** Meaningful within patient change estimates

Anchor	MMRM LS mean estimates (stable versus minimal improvement (95% CI) – Overall)	ROC (all improvement versus no change – Week)	Published thresholds	Proposed threshold based on current analysis*
PGIS	-0.41 (-0.54, -0.28)	-1.60 – Week 4 -0.80 – Week 8 -0.40 – Week 12	0.5[9]	0.6
PGIC	-0.24 (-0.44, -0.05)	-0.60 – Week 4		

Note: ROC Curves for PGIC Week 8 and Week 12 were not interpreted/reported because the AUC 95% CI lower bound was < 0.5

*Presented as an absolute value as it evaluates difference in scores

The Sum of Squares method was used to find the optimal MWPC threshold. 95% CIs were not produced for the ROC Curve PRO score cutoff estimates, so are not presented here

Only MMRM estimates where the effect size was between 0.2 and 2 were considered tenable. Estimates where the effect size was outside of this range were excluded

Estimates with a group size *n* < 10 were omitted

Abbreviations: CI = Confidence interval; LS = Least Squares; MMRM = Mixed models for repeated measures; PGIC=Patient Global Impression of Change; PGIS=Patient Global Impression of Severity; ROC = Receiver operating characteristic

#### Meaningful between-group difference

##### MBGD estimates

Estimates for the MBGD were generally consistent in magnitude and ranged from − 0.23 to − 0.56. The PGIC estimate (-0.23) was smaller in magnitude than all PGIS estimates (range: -0.41 to -0.56) (Table 6).

**Table 6:** Meaningful between-group difference estimates

Anchor	MMRM LS mean estimates – best health state versus next best health state (95% CI) - Overall	Group mean change - stable versus minimal improvement (95% CI) - Week	Published threshold	Optimal threshold range based on current study data*
PGIS	-0.55 (-0.70, -0.40)	-0.37 (-0.63, -0.12) – Week 4 -0.56 (-0.82, -0.29) – Week 8 -0.41 (-0.67, -0.14) – Week 12	0.5[9]	0.2 to 0.6
PGIC	Not applicable**	-0.23 (-0.51, 0.05) – Week 4		

Note: PGIC at Week 8 was not interpreted/reported as the estimate was positive (in the wrong direction) and for Week 12 because 0.00 was an untenable estimate

*The range is presented as an absolute value as it evaluates difference in scores

**PGIC change response format is not applicable to cross-sectional methodology

Only MMRM estimates where the effect size was between 0.2 and 2 were considered tenable. Estimates where the effect size was outside of this range were excluded

Estimates with a group size *n* < 10 were omitted

Abbreviations: CI = Confidence interval; LS = Least Squares; MMRM = Mixed models for repeated measures; PGIC=Patient Global Impression of Change; PGIS=Patient Global Impression of Severity

##### Proposed MBGD

The anchor-based estimates were triangulated and summarized on a forest plot to aid interpretation (Figure S6). The proposed threshold range is shown in Table 5. This range is reflective of the anchor-based estimates rounded up to one decimal place for ease of use. Notably, the range is inclusive of the previously published 0.5 threshold, thereby supporting its use while also suggesting that smaller magnitudes of change may still be meaningful to patients (i.e., the lower bound of the range).

#### Classification statistics analysis evaluating ACQ-5 ‘well-controlled’ asthma threshold

**Fig. 2:**
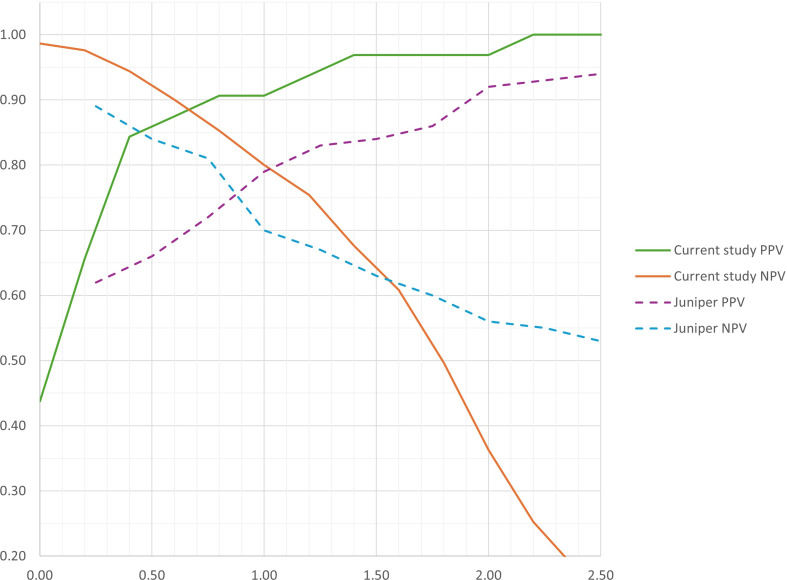
Plot of PPV and NPV values reported in Juniper et al. [21] and those derived using the Phase 2 trial data for ‘well-controlled’ and ‘not well-controlled’ participants according to ACQ-5 total score

Figure 2 shows the point at which the PPV and NPV derived in each study intersect (or are simultaneously maximized). The intersection of PPV and NPV in the present study was approximately ACQ-5 = 0.67, compared to Juniper et al. whose intersection was ACQ-5 = 0.88 [21]. A ‘well-controlled’ ACQ-5 threshold of ≤ 0.75 had an NPV of 0.86 and PPV of 0.90, which is consistent with the NPV supporting ≤ 0.75 in the original research and exceeds the PPV used to determine the threshold for “well-controlled” asthma (NPV = 0.81; PPV = 0.72) [21].

## Discussion

This study aimed to derive meaningful change thresholds using contemporary methods and evaluate the applicability of score interpretation and diagnostic classification thresholds established in previous research. Analyses were performed in accordance with best practices for assessing measurement properties of clinical outcome assessments [19, 27, 28]. Consistent with prior research conducted by the ACQ-5 developers, findings of this study supported use of a 0.5 threshold for MWPC and MBGD improvement, and a cross-sectional threshold of ≤ 0.75 to define ‘well-controlled’ asthma. Supportive evidence for various ACQ-5 measurement properties in a contemporary sample of adults with inadequately controlled moderate-to-severe asthma was also generated.

### Meaningful change thresholds

Evidence generated from the meaningful change analyses supports use of the previously established ≥ 0.5 threshold [9, 41]. This study provides updated evidence using contemporary score interpretation methods more aligned with current literature and regulatory guidance [15, 19]. Importantly, this study provides clarification and reassurance regarding whether the extant ≥ 0.5 threshold should be used as a between-group or within-individual threshold; this distinction was not typical at the time of the original research, which has led to ambiguity concerning application of the 0.5 threshold as a within-individual or between-group threshold [41]. Although results from this study suggest that a MWPC threshold of ≥ 0.6 improvement is supported for the ACQ-5, when considering the attainable scale increments of 0.2 for the ACQ-5 [20], an improvement of ≥ 0.6 is functionally identical to ≥ 0.5. Supportive distribution-based analyses also suggested a ≥ 0.6 threshold would exceed measurement error.

Our results were also consistent with the use of the existing ≥ 0.5 threshold as a MBGD threshold. However, rather than applying this as a sole point estimate, this study builds on previous work by providing a range of estimates (i.e., 0.2 to 0.6), as per contemporary recommendations relating to the application of MBGDs [19]. The ranges produced provide further context regarding smaller magnitudes of change that may still be meaningful to patients (i.e., the lower bound of the range).

### Asthma control thresholds

Exploration of classification statistics for the ACQ-5 change scores also provided general support for continued use of a ≤ 0.75 threshold to determine well-controlled asthma in the current data. Specifically, a ≤ 0.75 threshold resulted in a similar NPV (NPV = 0.86) to those originally calculated for the ACQ-5 (NPV = 0.81) by Juniper et al. [21].

### Measurement properties of the ACQ-5

Strong and moderate correlations between the ACQ-5 and measures of related concepts were observed at Week 12, providing evidence of convergent validity. Known-groups comparisons showed that the ACQ-5 scores differed in groups based on average reliever medication use; evidence discriminating clinically distinct groups is generally favored by the US FDA. Supportive evidence of known-groups validity was also observed based on groups formed by the PGIS. Improved participants showed larger improvements compared with stable participants, providing evidence that the ACQ-5 can detect improvements in asthma control according to both self-reported and physiological anchors.

Notably, test-retest reliability demonstrated only a modest level at post-Baseline timepoints and did not reach typically acceptable test-retest reliability thresholds from Baseline to Week 4. These results were likely influenced by aspects of the trial design that limited variance in the sample, as well as the ability to define a stable sample for this analysis (explained in detail in the study considerations section below).

Additionally, item response distributions demonstrated limited endorsement of the full response scale. Although formal ceiling effects were not observed at Baseline, responses were strongly skewed toward milder response options despite the inclusion of a moderate-to-severe sample. As patients improved over time, ceiling effects emerged at later assessment timepoints, indicating a continuation of this pattern. This reduction in score variability limits sensitivity to detect further clinical improvement. Given the episodic nature of asthma, the use of a weekly recall period may contribute to this skew. Substantial symptoms within a week may be averaged out in patient reports, attenuating responses. In recognition of this limitation, more contemporary instruments have adopted daily recall periods to better capture the short-term variability characteristic of asthma symptoms [42].

### Study considerations

Psychometric evidence is crucial for supporting treatment benefit by ensuring assessments included in clinical trials accurately and consistently measure what they intend to measure. This ensures that trial data meet regulatory standards (i.e., of high quality and suitable for evaluating trial outcomes) [19] and that any observed changes are an effect of treatment rather than methodological issues [43]. While the results of this study are supportive of the existing evidence for strong psychometric properties of the ACQ-5, some psychometric analyses were limited by aspects of the Phase 2 trial design.

The requirement for trial participants to have an ACQ-5 total score between 1.25 and 3.00 at randomization reduced between-subject variance in ACQ-5 scores at Baseline and later timepoints. Study participants who experienced a loss of asthma control event were also discontinued from the study, further reducing between-subject variance at later timepoints (i.e., the most severe participants were removed). As both between- and within-subject variance in data are crucial for ICC estimation [44], this inclusion and discontinuation criteria may have impacted test-retest reliability, resulting in weaker results than observed in previous research [7, 9, 45, 46]. This criteria may also explain why better reliability results were observed by Week 12, when participants’ scores were less restricted and naturally varied due to the course of disease. Test-retest reliability may also have been impacted by withdrawal of the ICS/LABA treatment phase and lack of a concept specific PGIS for asthma control, limiting ability to define a sufficiently-sized stable sample for this analysis.

The results of this study suggest that consideration should be given to criteria that may have restricted the range (variance) of scores in a sample. In the context of this study, the ACQ-5 entry criteria (or lack of an alternative measure to determine study eligibility), the length of the screening period, and/or exclusion of participants who experienced exacerbations could have restricted variance in the sample, thereby impacting the precision of reliability estimates [47]. Further, although inclusion of PGI items in this study is a strength compared with earlier psychometric evidence of the ACQ-5 [9], current regulatory guidance recommends the use of anchor measures that closely match the specific concept of a target measure [19]; in this case, asthma control. Investigators planning to use the ACQ-5 in future clinical studies should ensure that PGI items are designed to assess asthma control to maximize the potential strength of anchor correlations [48, 49].

Results are also limited by composition of the study sample. As nearly all participants were White (95.4%) and only adults with moderate-to-severe uncontrolled asthma were included in the study cohort, conclusions may not be generalizable to other racial or ethnic groups or those with mild asthma.

### Recommendations for future research

To further expand the generalizability of the results, future research should conduct large-scale, multi-center validation studies covering diverse racial and ethnic groups and the full spectrum of asthma severity. Additionally, sufficient sample sizes should be pre-specified to support sub-group analyses; specifically, those experiencing asthma exacerbations to support robust assessment of responsiveness to symptom worsening. Asthma-control specific PGI anchors should also be used to further improve the level of evidence for the derived thresholds.

## Conclusions

Contemporary score interpretation methods support a MWPC threshold of ≥ 0.6, which is functionally identical to the previously established threshold of ≥ 0.5. Use of the extant ≥ 0.5 threshold as a MBGD threshold was also supported, with results suggesting magnitudes of change between 0.2 and 0.6 as likely meaningful to patients. Applicability of the ≤ 0.75 threshold for defining “well-controlled” asthma was also confirmed. Psychometric analyses from this study support previous research by demonstrating acceptable measurement properties of the ACQ-5 for use in a moderate-to-severe asthma population.

## Electronic Supplementary Material

Below is the link to the electronic supplementary material.

Supplementary Material 1

## Data Availability

The datasets generated and/or analyzed during the current study are available from the corresponding author at reasonable request.

## Abbreviations

ACQ-5: Asthma control questionnaire-5 items
ADSD: Asthma daytime symptom diary
ANSD: Asthma nighttime symptom diary
ATS: American Thoracic Society
BD: Bronchodilator
BID: Twice a day
COA: Clinical outcome assessment
eCDF: Empirical cumulative distribution function
ERS: European respiratory society
ES: Effect size
FEV: Forced expiratory volume
ICC: Intraclass correlation coefficient
ICS: Inhaled corticosteroids containing medication
LABA: Long-acting beta-agonist
LS: Least squares
MBGD: Meaningful between-group difference
MWPC: Meaningful within-person change
MMRM: Mixed models for repeated measures
NPV: Negative predictive value
PEF: Peak expiratory flow
PGIC: Patient global impression of change
PGIS: Patient global impression of severity
POC: Proof-of-concept
PPV: Positive predictive value
PRO: Patient-reported outcome
ROC: Receiver operating characteristic
SD: Standard deviation
SDC: Smallest detectable change
SEM: Standard error of measurement
TID: Three times a day

## Footnotes

1 LOAC event was defined as a ≥ 30% reduction from baseline in morning peak expiratory flow (PEF) on 2 consecutive days; ≥6 additional reliever puffs of salbutamol/albuterol or levosalbutamol/levalbuterol in a 24-hour period (compared to baseline) on 2 consecutive days; an increase in ICS ≥ 4 times the last prescribed dose (or ≥ 50% at visit 2 if background therapy withdrawal was completed); use of systemic steroid treatment; or hospitalization/emergency room visit for exacerbation.
