# Supplementary material for: Justifying score interpretation thresholds for the Asthma Control Questionnaire (ACQ-5) in a contemporary asthma patient population
Source: J Patient Rep Outcomes. 2026 May 30;10:133. doi: 10.1186/s41687-026-01097-y (PMC13442793; doi:10.1186/s41687-026-01097-y)
Supplement: Supplementary file 1 [file 41687_2026_1097_MOESM1_ESM.docx]

| Table S1. Definitions used to determine well-controlled vs. uncontrolled asthma | | | | | |
| --- | --- | --- | --- | --- | --- |
| Asthma symptom control | | | | | |
| **Asthma control definition according to GINA guidelines** | | | | | |
| **In the past 4 weeks, has the patient had:** | | | **Well controlled** | **Partly controlled** | **Un-controlled** |
| 1. Daytime asthma symptoms more than twice/week? | Yes🞏 | No🞏 | None of these | 1-2 of these | 3-4 of these |
| 2. Any night waking due to asthma? | Yes🞏 | No🞏 |  |  |  |
| 3. SABA reliever for symptoms more than twice/week? | Yes🞏 | No🞏 |  |  |  |
| 4. Any activity limitation due to asthma? | Yes🞏 | No🞏 |  |  |  |
| **Proxy items used to assess asthma control based on GINA guidelines** | | | | | |
| 1. Response >0 on any ADSD item for more than two days/week | | | | | |
| 2. Response <7 to Item 24 on AQLQ[S] Activity limitation domain | | | | | |
| 3. Reliever medication reported in patient-reported eDiary completion | | | | | |
| 4. Response <7 to any item on the AQLQ[S] Activity limitation domain | | | | | |

| Table S2. Internal Consistency Reliability: Cronbach's Alpha and Alpha-if-removed for the ACQ-5 score for the Psychometric Analysis Population at Baseline and Week 12 in the psychometric analysis population | | |
| --- | --- | --- |
|  | **Baseline (n=196)** | **Week 12 (n=138)** |
| Cronbach's alpha (Total) | 0.61 | 0.90 |
| **Cronbach's alpha if item deleted** | | |
| Item 1: Frequency of nighttime awakenings | 0.57 | 0.89 |
| Item 2: Severity of symptoms upon waking | 0.52 | 0.86 |
| Item 3: Severity of impact on daily activities | 0.60 | 0.88 |
| Item 4: Severity of shortness of breath | 0.52 | 0.86 |
| Item 5: Frequency of wheeze | 0.55 | 0.89 |

| Table S3. Convergent correlations for the ACQ-5 with other relevant measures at Baseline and Week 12 in the psychometric analysis population | | | | |
| --- | --- | --- | --- | --- |
| **Convergent measure** | **Coefficient type** | **Hypothesized magnitude** | **Baseline (n=196)** | **Week 12 (n=138)** |
| PGIS | Polyserial | >0.50 | 0.41 | 0.83 |
| ADSD | Spearman | >0.50 | 0.45 | 0.59 |
| ANSD | Spearman | >0.50 | 0.46 | 0.52 |

| Table S4. Known-groups comparisons for the ACQ-5 at Baseline and Week 12 in the psychometric analysis population | | | | | | |
| --- | --- | --- | --- | --- | --- | --- |
| Known-groups anchor | Timepoint | N | Mean (SD) | Median | One-way ANOVA P-value* | Between-groups effect size** |
| PGIS | | | | | | |
| None | Baseline | 7 | 2.0 (0.44) | 2.2 | - | - |
| Mild |  | 70 | 2.0 (0.33) | 2.0 | - | 0.16 |
| Moderate |  | 109 | 2.3 (0.37) | 2.2 | - | 0.80 |
| Severe |  | 6 | 2.5 (0.45) | 2.5 | <0.001 | 0.56 |
| None | Week 12 | 24 | 0.4 (0.45) | 0.4 | - | - |
| Mild |  | 81 | 1.4 (0.58) | 1.4 | - | 1.84 |
| Moderate |  | 30 | 2.2 (0.72) | 2.1 | - | 1.33 |
| Severe |  | 3 | 4.1 (1.17) | 3.6 | <0.001 | 2.42 |
| PGIS – collapsed categories | | | | | | |
| None or mild | Baseline | 77 | 2.0 (0.33) | 2.0 | - | - |
| Moderate or severe |  | 115 | 2.3 (0.37) | 2.2 | <0.001 | 0.81 |
| None | Week 12 | 24 | 0.4 (0.45) | 0.4 | - | - |
| Mild |  | 81 | 1.4 (0.58) | 1.4 | - | 1.84 |
| Moderate or severe |  | 33 | 2.4 (0.92) | 2.2 | <0.001 | 1.43 |
| Reliever medication | | | | | | |
| Mean inhalations <1/day | Baseline | 127 | 2.2 (0.42) | 2.2 | - | - |
| Mean inhalations ≥1/day |  | 66 | 2.2 (0.44) | 2.2 | 0.378 | 0.13 |
| Mean inhalations <1/day | Week 12 | 103 | 1.3 (0.80) | 1.4 | - | - |
| Mean inhalations ≥1/day |  | 25 | 2.0 (0.78) | 1.8 | <0.001 | 0.96 |
| *Abbreviations:* PGIC=Patient Global Impression of Change; PGIS=Patient Global Impression of Severity; SD=Standard Deviation  Well controlled asthma = 0 to 0.75, partially controlled asthma = 0.75 to 1.50 and uncontrolled asthma = >1.50.  * p-value is based on ANOVA model with PGIS categories variable as covariate  ** Cohen’s d effect size is calculated as the mean difference in PRO scores between adjacent PGIS categories divided by the pooled standard deviation of those categories.  Note: due to the small number of participants categorized as ‘severe’ at Week 12, the models were run based on the original PGIS groupings and based on combining the ‘moderate’ and ‘severe’ groups. | | | | | | |

Percentage of participants (%)

|  | 0-no impairment | 1 | 2 | 3 | 4 | 5 | 6-maximum impairment |
| --- | --- | --- | --- | --- | --- | --- | --- |
| Item 1: Frequency of nighttime awakenings | 3.1 | 21.9 | 61.7 | 12.8 | 0.5 | 0 | 0 |
| Item 2: Severity of symptoms upon wakening | 0 | 6.6 | 53.1 | 38.8 | 1.5 | 0 | 0 |
| Item 3: Severity of impact on daily activities | 1 | 9.2 | 62.8 | 26 | 1 | 0 | 0 |
| Item 4: Severity of shortness of breath | 0.5 | 4.6 | 53.6 | 37.8 | 3.6 | 0 | 0 |
| Item 5: Frequency of wheeze | 0.5 | 18.9 | 53.1 | 23 | 4.1 | 0.5 | 0 |

**Figure S1. Item response distributions for the ACQ-5 at Baseline in the psychometric analysis population (n=196)**

Percentage of participants (%)

|  | 0-no impairment | 1 | 2 | 3 | 4 | 5 | 6-maximum impairment |
| --- | --- | --- | --- | --- | --- | --- | --- |
| Item 1: Frequency of nighttime awakenings | 24.7 | 27 | 34.3 | 13.5 | 0.6 | 0 | 0 |
| Item 2: Severity of symptoms upon wakening | 14 | 24.7 | 32 | 27 | 2.2 | 0 | 0 |
| Item 3: Severity of impact on daily activities | 18 | 23 | 39.9 | 16.3 | 2.8 | 0 | 0 |
| Item 4: Severity of shortness of breath | 12.9 | 23.6 | 33.7 | 25.8 | 3.4 | 0.6 | 0 |
| Item 5: Frequency of wheeze | 16.3 | 27.5 | 31.5 | 21.3 | 2.2 | 1.1 | 0 |

**Figure S2. Item response distributions for the ACQ-5 at Week 4 in the psychometric analysis population (n=178)**

Percentage ofparticipants (%)

|  | 0-no impairment | 1 | 2 | 3 | 4 | 5 | 6-maximum impairment |
| --- | --- | --- | --- | --- | --- | --- | --- |
| Item 1: Frequency of nighttime awakenings | 23.8 | 33.1 | 29.4 | 11.3 | 1.9 | 0.6 | 0 |
| Item 2: Severity of symptoms upon wakening | 13.1 | 30.6 | 35 | 18.1 | 1.9 | 1.3 | 0 |
| Item 3: Severity of impact on daily activities | 18.8 | 32.5 | 35 | 10 | 1.9 | 1.9 | 0 |
| Item 4: Severity of shortness of breath | 14.4 | 28.1 | 35.6 | 15.6 | 4.4 | 1.3 | 1 |
| Item 5: Frequency of wheeze | 20.6 | 29.4 | 28.8 | 13.8 | 5.6 | 1.9 | 0 |

**Figure S3. Item response distributions for the ACQ-5 at Week 8 in the psychometric analysis population (n=160)**

Percentage of participants (%)

|  | 0-no impairment | 1 | 2 | 3 | 4 | 5 | 6-maximum impairment |
| --- | --- | --- | --- | --- | --- | --- | --- |
| Item 1: Frequency of nighttime awakenings | 29.7 | 31.9 | 30.4 | 5.1 | 2.2 | 0.7 | 0 |
| Item 2: Severity of symptoms upon wakening | 17.4 | 27.5 | 34.8 | 15.9 | 3.6 | 0 | 0.7 |
| Item 3: Severity of impact on daily activities | 23.9 | 32.6 | 31.2 | 9.4 | 2.2 | 0.7 | 0 |
| Item 4: Severity of shortness of breath | 14.5 | 28.3 | 39.9 | 15.2 | 0.7 | 0.7 | 0.7 |
| Item 5: Frequency of wheeze | 23.2 | 26.1 | 37.7 | 6.5 | 5.1 | 1.4 | 0 |

**Figure S4. Item response distributions for the ACQ-5 at Week 12 in the psychometric analysis population (n=138)**

Abbreviations: AUC = Area under the curve; MMRM = Mixed Models for Repeated Measures; PGIC = Patient Global Impression of Change; PGIS = Patient Global Impression of Severity; ROC = Receiver Operating Characteristic; SD = Standard Deviation; SDC = Smallest Detectable Change; SEM = Standard Error of Mean; r_avg = Average Correlation

Figure S5. Forest plot showing estimated meaningful within-patient change thresholds for the ACQ-5 in the psychometric analysis population

Abbreviations: MMRM = Mixed Models for Repeated Measures; PGIC = Patient Global Impression of Change; PGIS = Patient Global Impression of Severity; SD = Standard Deviation; SEM = Standard Error of Mean; r_avg = Average Correlation

Figure S6. Forest plot showing estimated between-group meaningful change thresholds for the ACQ-5 in the psychometric analysis population
